# 2,4-Diacetylphloroglucinol Modulates *Candida albicans* Virulence

**DOI:** 10.3390/jof8101018

**Published:** 2022-09-27

**Authors:** Artyom A. Stepanov, Darya V. Poshvina, Alexey S. Vasilchenko

**Affiliations:** Laboratory of Antimicrobial Resistance, Institute of Environmental and Agricultural Biology (X-BIO), Tyumen State University, Volodarskogo Street, 6, 625003 Tyumen, Russia

**Keywords:** 2,4-diacetylphloroglucinol, *Candida albicans*, biofilm, virulence, aspartyl protease, hyphal channels

## Abstract

The dimorphic fungus *Candida albicans* is one of the most important opportunistic pathogens for humankind. The use of fungicides against *Candida* could be associated with sub-inhibitory effects, which are referred to as fungal stress responses and are undesirable for the host. In this work, we investigated the antifungal action of 2,4-diacetylphloroglucinol (2,4-DAPG) against *Candida albicans* ATCC 10231 with a focus on their biofilm-forming ability. We found that 2,4-DAPG was able to reduce the ability of *Candida* cells to form biofilms, but complete inhibition and eradication effects were not achieved. Furthermore, *C. albicans* cells in the adherent state were characterized by reduced susceptibility to 2,4-DAPG compared to planktonic cells. The investigation of the mechanisms that could explain the antibiofilm action of 2,4-DAPG revealed a reduction in the cell`s surface hydrophobicity and the inhibition of the yeast-to-hyphae transition. The inhibition of the *Candida* cells filamentation was accompanied by an increase in the expression of the *NRG1* gene, which is a negative regulator of hyphal development. In addition, we microscopically visualized the treated biofilms and revealed numerous channels that were decorated with particles and localized on the hyphae. We assumed that these hyphal structures could be associated with the secretion of aspartyl proteases (Sap). The performed assessments revealed an increase in the activity of Sap, which was accompanied by an increase in the expression of the *sap2* and *sap4* genes. The antifungal action of 2,4-DAPG is known to be associated with affecting the permeability of cellular structures, which leads to H^+^ATPase malfunction and the disruption of mitochondrial respiration. The subsequent cytosol acidification and generation of ROS trigger the inhibition of *Candida* filamentation and activation of Sap production. The introduction of antioxidant Trolox simultaneously with 2,4-DAPG leads to a reduction in Sap production. Collectively, the obtained data indicate new aspects of the interaction of fungal cells with 2,4-DAPG, an antimicrobial metabolite of *Pseudomonas* spp.

## 1. Introduction

2,4-diacetylphloroglucinol (2,4-DAPG) is a small molecule produced as a secondary metabolite by *Pseudomonas* spp. [1]. The genes which are involved in the production of 2,4-DAPG and other phloroglucinol derivatives are very common among various species of *Pseudomonas* genus [2]. 2,4-DAPG has the ability to kill a wide range of microorganisms, including bacteria and fungi. It is known that the antimicrobial effect of 2,4-DAPG against fungi is caused by the uncoupling of respiration and ATP synthesis in fungal cells by disrupting membrane integrity [3,4]. 

The indirect effect of 2,4-DAPG is the disorganization of fungal metabolism. For example, the production of fusaric acid by *Fusarium oxysporum* was dysregulated at sub-inhibitory concentrations [5]. Moreover, the indirect antimicrobial effect of 2,4-DAPG could be associated with its activity as a signal messenger. For example, it is reported that 2,4-DAPG can interfere with the expression of genes involved in the biofilm formation of *Bacillus subtilis* [6], and it can inhibit the production of quorum-sensing autoinducer by *Pectobacterium carotovorum* [7].

Despite the fact that the antimicrobial properties of 2,4-DAPG have been known for a long time, the available works revealed its effect on planktonic cells only. At the same time, microorganisms, including fungi, generally exist in a biofilm form. Previously, we have shown the anti-biofilm potential of 2,4-DAPG against bacterial biofilms [7], while 2,4-DAPG action against fungal biofilms has not been tested yet.

*Candida albicans* is an opportunistic human pathogen that frequently causes superficial infections but, in some cases, may cause systemic infections [8]. The virulence of *C. albicans* is associated with the ability to persist in a biofilm state and produce various enzymes, which allows yeasts to disseminate within the host`s tissue [9]. The biofilm of *C. albicans* is a structured population of sessile yeast cells with pseudo-hyphae and hyphae, which are surrounded by an exopolymeric matrix [8]. The biofilm allows *C. albicans* to be resistant to antifungals and remain hidden from the host’s immune system. Biofilms on medical devices are an increasing problem in healthcare. Moreover, there is a limited number of antifungals available for therapy (e.g., amphotericin B, fluconazole), which, along with that, possess high cytotoxicity for a host [10].

In this paper, we challenged the antifungal activity of 2,4-DAPG using the model yeast pathogen *Candida albicans* ATCC 10231. The aim of this work was to determine the ability of 2,4-DAPG to affect yeast metabolism and prevent biofilm formation. The 2,4-DAPG action was evaluated towards forming and mature biofilms with subsequent analysis of the underlying mechanisms. We focused on the phenotypical and genetic differences between the intact and treated *Candida* biofilms. We established the important morphological changes with *Candida* hyphae, which had not been described earlier. The revealed hyphal changes could be associated with aspartyl protease excretion and are the manifestation of the sub-inhibitory effect of 2,4-DAPG on *C. albicans*. 

## 2. Materials and Methods

### 2.1. Strain and Media

*C. albicans* ATCC 10231 was used as a model fungal microorganism. Before assays, *C. albicans* was cultured on YPD agar (1% yeast extract, 2% peptone, 2% dextrose, 1.5% agar) at 37 °C for 24 h. Prior to each assay, colonies of *C. albicans* were transferred in YPD broth and incubated at 30 °C overnight with an agitation of 100 rpm.

The required amount (10 mg) of 2,4-DAPG (Abcam, Cambridge, UK) was weighed and dissolved in 1 mL of DMSO (99.99 % purity, ApplyChem, Darmstadt, Germany).

### 2.2. Determination of Minimum Inhibitory Concentration 

The determination of the minimum inhibitory concentration (MIC) was performed following the Clinical and Laboratory Standards Institute guidelines for yeast broth dilution antifungal susceptibility testing [11,12]. The fungal growth was assessed by measuring the absorbance data at 620 nm using a spectrophotometer (Multiscan GO, Thermo Fisher Scientific, Waltham, MA, USA). The MIC was determined to be the lowest concentration of antibiotic for which no visible fungal growth could be observed after 24 h of incubation.

### 2.3. Biofilm Inhibition and Biofilm Destruction Assays

The inhibitory effect of 2,4-DAPG on *C. albicans* biofilms was investigated by the development of biofilms on polystyrene flat bottom 96-well microtiter plates, according to Khan et al. [13], with some modifications.

Following the adhesion phase (i.e., 10^7^ cells incubated in 100 μL RPMI-1640 with L-glutamine and phenol red (Sigma-Aldrich, Steinheim, Germany) for 90 min at 75 rpm at 37 °C), the cell suspensions were aspirated, and each well was washed twice with 150 μL of phosphate-buffered saline (PBS) (P4417 catalog No, Sigma-Aldrich, St. Louis, MO, USA) to remove loosely adherent cells. RPMI-1640 medium containing 2,4-DAPG was transferred to each of the washed wells up to the final volume of 100 μL. The solution of DMSO (5% *v*/*v*) in the RPMI-1640 medium was taken as the control sample.

The plates were incubated at 37 °C on a shaker at 75 rpm for 24 and 48 h, and the culture medium was replenished daily. The metabolic activity of the *C. albicans* biofilms was determined quantitatively using the 2,3,5-triphenyl-tetrazolium chloride (TTC) reduction assay [14]. 

Briefly, the solution (1%) of 2,3,5-triphenyl-tetrazolium chloride (TTC) (DiaM, Moscow, Russia) was prepared by dissolving TTC in distilled water, then sterilized by filtration through 0.22 μm PVDF filters (Millipore, Burlington, VT, USA). Then, 25 μL of the TTC solution and 75 μL of the YPD were added to the wells of microtiter plates containing *C. albicans* biofilms. The plates were incubated in the dark for 3 h at 37 °C. After incubation, the supernatant was removed, and 96% ethanol was added. Dissolved formazan was transferred to a new 96-well microplate, and then the absorbance was measured at 490 nm.

The total biomass of the biofilms was visualized using a crystal violet assay [15]. Briefly, the *C. albicans* biofilms developed in 96-well microplates were twice washed with 200 μL PBS, then air dried for 45 min. After that, the wells were stained with 110 μL of 0.2% aqueous crystal violet solution for 15 min. Afterward, the wells were washed three times with 300 μL of sterile distilled water and subsequently discolored with 200 μL of 95% ethanol. After 15 min, 100 μL of ethanol solution was transferred to a new 96-well microplate, and then the absorbance was measured at 590 nm using a spectrophotometer Multiscan GO (Thermo Fisher Scientific, Waltham, MA, USA). Biofilm inhibition was calculated using the formula:

Biofilm inhibition (%) = ((OD_control_ − OD _treated_)/OD_control_) × 100

where OD is the absorbance value. 

### 2.4. The Effect of 2,4-DAPG on Pre-Formed Biofilms

The *C. albicans* biofilms were prepared for 24 h at 37 ºC in a shaker at 75 rpm as described above. The wells of the microtiter plates were washed twice with PBS and filled with fresh RPMI-1640 medium (100 µL) containing different concentrations of 2,4-DAPG. The plates were placed in the bags with wet paper and incubated at 37 °C in a shaker at 75 rpm up to 48 h. The solution of DMSO (5% *v*/*v*) in the RPMI-1640 medium was taken as the control sample. The metabolic activity of the *C. albicans* biofilms and the total biomass of biofilms were determined quantitatively, as described above.

### 2.5. The Effect of 2,4-DAPG on Germ Tube Formation

The germ tube inhibition assay was performed in 96-well microplates in accordance with Zuzarte et al. [16] with some modifications. The N-acetyl-D-glucosamine medium was prepared as follows: 0.5% N-acetyl-D-glucosamine (Sigma-Aldrich, St. Louis, MO, USA), 0.5% peptone (Sigma-Aldrich, St. Louis, MO, USA), and 0.3% KH_2_PO_4_ were dissolved in distilled water at a final volume of 100 mL and filtered through PVDF-membrane with pores 0.22 µm (Millipore, Burlington, MA, USA). The inoculum suspension was adjusted to 10^7^ CFU / ml in PBS, then added to 170 µL of N-acetyl-D-glucosamine medium containing 2,4-DAPG. The wells without 2,4-DAPG were taken as negative controls. The microplates were incubated at 37 °C at 150 rpm for 3 h. 

After that, at least 100 yeast cells from each well in three technical repetitions were counted using a Primo Star microscope (Zeiss, Oberkochen, Germany). The inhibition of the germ tubes was calculated using the formula of Okamoto et al. [17]:

$$\mathrm{Germ}\;\mathrm{tube}\;\mathrm{formation}\;(\%)=\frac{\mathrm{Number}\;\mathrm{of}\;\mathrm{germ}\;\mathrm{tube}-\mathrm{forming}\;\mathrm{cells}}{\mathrm{Number}\;\mathrm{of}\;\mathrm{observed}\;\mathrm{cells}}\;\times \;100$$

To evaluate the inhibition of germ tubes on a solid medium, 10 µL of the inoculum suspension of *C. albicans*, adjusted to 10^6^ CFU/mL, was spotted on Spider agar plates containing different concentrations of 2,4-DAPG and control antimicrobials (amphotericin B (Sigma-Aldrich, St. Louis, MO, USA) and chlorhexidine bigluconate). The plates were incubated for 4 days at 37 °C, then the morphology of the fungal colonies was observed using a stereomicroscope and photographed using a digital camera.

### 2.6. Adhesion of C. albicans to Polystyrene

*C. albicans* was adhered to polystyrene using flat-bottom 96-well microtiter plates (Eppendorf, Hamburg, Germany) according to Vargas-Blanco et al. [18] with some modifications. *C. albicans* ATCC 10231 was grown overnight in YPD broth. The fungal cells were harvested; afterwards, they were washed twice with PBS and adjusted to 10^7^ CFU/mL. The prepared inoculum was used for inoculation in a 96-well microtiter plate containing RPMI-1640 medium and different concentrations of 2,4-DAPG. RPMI-1640 medium with 5% DMSO was included as the control. The plates were incubated for 90 min at 37 °C. Following incubation, non-adherent *C. albicans* cells were removed by aspiration. The metabolic activity and the total biomass of adherent cells were determined quantitatively using the 2,3,5-triphenyl-tetrazolium chloride (TTC) reduction assay and the crystal violet assay, respectively.

### 2.7. Hydrophobicity of C. albicans Cell Surface

Cell surface hydrophobicity (CSH) was assessed using the microbial adhesion assay to hydrocarbons according to Salva-Dias et al. [19] with some modifications. *C. albicans* was grown overnight at 37 °C, then harvested and washed twice with PBS. Cell suspension with optical density 0.5–0.6 (λ = 600 nm) was prepared in PBS (A_0_); 2 mL of yeast suspension was overlaid by 0.3 mL of the hydrophobic hydrocarbon, n-hexane. After vigorous vortexing (1 min), the phases were allowed to separate (10 min) at 30 °C, and the optical density at 600 nm of the aqueous phase was measured (A_1_). The percentage of hydrophobicity was calculated by the formula:

hydrophobicity (%) = (1 − (A_1_/A_0_)) × 100

### 2.8. Aspartyl Protease Activity of C. albicans Planktonic Cells and Biofilms 

The production of aspartyl protease (Sap) of the 1–3 subfamily was determined according to Jothi et al. [20] with some modifications. Briefly, the *C. albicans* planktonic cells and biofilms were grown in a YPD medium supplemented with inhibitory or/and sub-inhibitory concentrations of 2,4-DAPG for 24 h. After incubation, the cell-free supernatant was collected by centrifugation at 8000× *g* rpm for 10 min. Then, 0.1 mL of supernatant was mixed with 0.9 mL of 50 mM citrate buffer (pH 3.5) consisting of 0.2% bovine serum albumin (BSA). After incubation at 37 °C for 15 min, the mixture was precipitated with 0.5 mL 15% trichloroacetic acid (TCA) by centrifugation at 10,000× *g* rpm (4 °C) for 5 min. The concentration of soluble products was measured at 280 nm. 

The production of the Sap4–6 subfamily was measured according to Tamura et al. [21] with some modifications. Briefly, 0.15 mL of cell-free supernatant was combined with 0.25 mL of 1% azocasein in 50 mM Tris-HCl buffer (pH 5.5) and incubated at 37 °C for 1 h. The reaction was stopped by adding 0.4 mL of TCA (10%). Subsequently, the supernatant was harvested by centrifugation at 10,000× *g* rpm (4 °C) for 10 min, then was combined with an equal volume of 0.5 M NaOH and incubated at 37 °C for 15 min. The concentration of the soluble products was measured at 440 nm.

### 2.9. Lipase Activity of C. albicans

The lipase activity was measured according to Ravindran et al. [22] with some modifications. Briefly, *C. albicans* was grown in a YPD medium supplemented with 1% Tween-20 and 2,4-DAPG at concentrations of 7.5, 15, 30, and 60 µg/mL for 24 h. After incubation, the cell-free supernatant was collected by centrifugation at 8000× *g* rpm for 10 min. Then, 0.1 mL of supernatant was mixed with 0.9 mL of substrate, which contained 1 volume of 0.3% p-nitrophenyl palmitate (pNPP) in isopropanol and 9 volumes of 0.2% Triton X-100 and 0.1% gummi arabicum in 25 mM Tris-HCl buffer (pH 8.0). The samples were incubated for 15 min at 25 °C. After incubation, the reaction was terminated by adding 0.5 mL of 1 M Na_2_CO_3_. The absorbance of the solution was measured at 410 nm.

### 2.10. Chemical Analysis of Biofilm Matrix Material

The extrapolymeric substance (EPS) from *C. albicans* biofilms was extracted using the formaldehyde/NaOH method [23]. Briefly, the *C. albicans* biofilms were scrapped from microtiter wells, resuspended in PBS, and vortexed at 300 rpm for 20 min. The biofilm suspension was mixed with formalin at a ratio of 1:160 for 1 h, 4 °C; then NaOH (1 M, 4 °C) was added with incubation for 3 h. The samples were centrifuged at 15,000 rpm for 25 min (4 °C), and the obtained supernatants were filtered through a 0.22 µm membrane. The filtrates were used as EPS samples. The proteins were purified with a dialysis membrane (5 kDa) at 4 °C for 24 h. The purified filtrate was frozen in liquid nitrogen and lyophilized. The freeze-dried pellet was resuspended in PBS. The carbohydrate content was measured with the phenol-sulphuric method, and the protein content was measured using NanoPhotometer N120 (Implen, Munich, Germany).

### 2.11. Microscopy Investigations of C. albicans Biofilms

**Scanning electron microscopy.** The *C. albicans* biofilms were prepared on an 8-well chamber slide (Eppendorf, Hamburg, Germany), according to Tsang et al. [24], with some modifications. Briefly, the slides were washed twice with PBS, dehydrated in a series of ethanol solutions (20% for 10 min, 50% for 10 min, 70% for 10 min, and 96% for 15 min), and dried overnight in a lyophilizer (at 1.5 mbar) prior to sputter-coating with gold. The surface topographies of the *C. albican*s biofilms were viewed with a MIRA3 LMU scanning electron microscope (Tescan, Brno, Czech Republic).

**Atomic force microscopy.** The biofilms were formed for the SEM investigation, and drying was performed under air for 12 h. The atomic force microscopy was performed using Integra NT-MDT (NT-MDT, Zelenograd, Russia) in the tapping mode. The microscopy investigation was carried out using the NSG01 cantilever (Tipsnano, Tallinn, Estonia) with a spring constant of ~5.1 N/m. Preliminarily, wide scanning of about 70 × 70 μm was conducted to produce a reference map of the sample surface in order to localize the yeast cells. The scan size was then set with a sampling of 512 by 512 points and a scan rate of 0.5 Hz. The topographic, amplitude and phase images were acquired simultaneously.

### 2.12. RNA Extraction and Real-Time Quantitative PCR Analysis 

The total RNA was isolated using the RNeasy Mini kit (Qiagen, Germantown, PA, USA) according to the manufacturer’s instructions. The total RNA was further treated with DNase I (New England Biolabs, Ipswich, MA, USA), followed by the RNA Clean and Concentrator-5 kit (Zymo Research, Irvine, CA, USA) according to the manufacturer’s instructions. The RNA was quantified using a Qubit RNA HS Assay Kit (Thermo Fisher Scientific, Waltham, MA, USA). The complementary DNA was obtained using the iScript gDNA clear cDNA synthesis kit (Bio-Rad, Hercules, CA, USA) in accordance with the manufacturer’s protocol. Then quantitative PCR was carried out using SsoAdvanced Universal SYBR Green Supermix reagent (Bio-Rad, Hercules, CA, USA). Each reaction mix contained (20 µL) 300 nM of the primer (final concentration) and 100 ng of the template RNA. 

The LightCycler96 Real-Time PCR detection system (Roche, Basel, Switzerland) was used with the following thermal cycling conditions: denaturation at 95 °C for 2 min, followed by 40 cycles of denaturation at 95 °C for 10 s and annealing/extension at 60 °C for 15 s. After the last amplification cycle, melting curve analysis was carried out by heating from 65 to 95 °C in increments of 0.5 °C/s. The negative controls (without a template or reverse transcriptase enzyme) were included in each run. Gene-specific primers (described in Table S1) were designed using the online service “Integrated DNA technologies” (https://www.idtdna.com/Primerquest/Home/Index [assessed on 20 July 2022]) using gene sequences presented in the GenBank database. The fold changes in the gene expression levels were normalized in relation to the levels of the gene encodes actin *(ACT1)* mRNA (Table S1). The relative changes in gene expression were quantified using the Pfaffl method [25]:

gene expression ratio = (E_target_) ^∆Ct target (control − sample)^/(E_reference_) ^∆Ct reference (control − sample)^,

where E_target_ is the amplification efficiency of target (gene of interest); E_reference_ is the amplification efficiency of reference (*ACT1*); Ct is the point at which the fluorescence rises above the background fluorescence; ∆Ct target is the Ct deviation of the control minus the sample of the target gene transcript; and ∆Ct reference is the Ct deviation of the control minus the sample of the reference gene transcript.

### 2.13. Quantitative Estimation of Tyrosol Production

*C. albicans* was grown in YPD at 30 °C, 110 rpm for 24 h. The cells were harvested by centrifugation at 9000× *g* for 25 min. The supernatant was filtered through a 0.22 µm membrane. Tyrosol was isolated from culture supernatants by solid-phase extraction, according to Alem et al. [26], with slight modifications. Tyrosol was quantified by reverse phase high-pressure liquid chromatography (RP-HPLC). The analytical standard of tyrosol was purchased from Sigma-Aldrich (St. Louis, MO, USA).

Prior to extraction, 100 mL of cell-free supernatant was acidified by adding 0.4 mL of 0.1 M H_2_SO_4_. The extraction was performed with a Strata C-18 cartridge (Phenomenex, Torrance, CA, USA). 

To quantify the amount of tyrosol in the extracts, the analytical RP-HPLC was performed using Luna C-18 analytical column (4.6 × 250 mm, 5 µm, Phenomenex, Torrance, CA, USA). The elution was carried out using solvent B (80% acetonitrile in ultrapure water with 0.1% TFA) in a linear gradient according to the following scheme: 0–5% for 5 min, 5–10% for 5 min, 10% for 5 min, 10–20% for 20 min, 20–70% for 5 min at a flow rate of 1.0 mL/min. The absorbance was detected at 225 nm. OpenLab CDS ChemStation software was used for processing the HPLC chromatograms. The calibration curve of tyrosol concentrations was plotted with 10, 20, 30, 50, and 100 µM of tyrosol.

### 2.14. Statistical Processing

The obtained results were statistically manipulated using Origin 2021 (OriginLab Corporation, Northampton, MA, USA) software. The Shapiro–Wilk test was used to assess the normality of value distributions. In the presence of a normal distribution, the pair-sample Student’s *t*-test was used. 

## 3. Results

### 3.1. Anti-Biofilm Activity of 2,4-DAPG 

**Figure 1:**
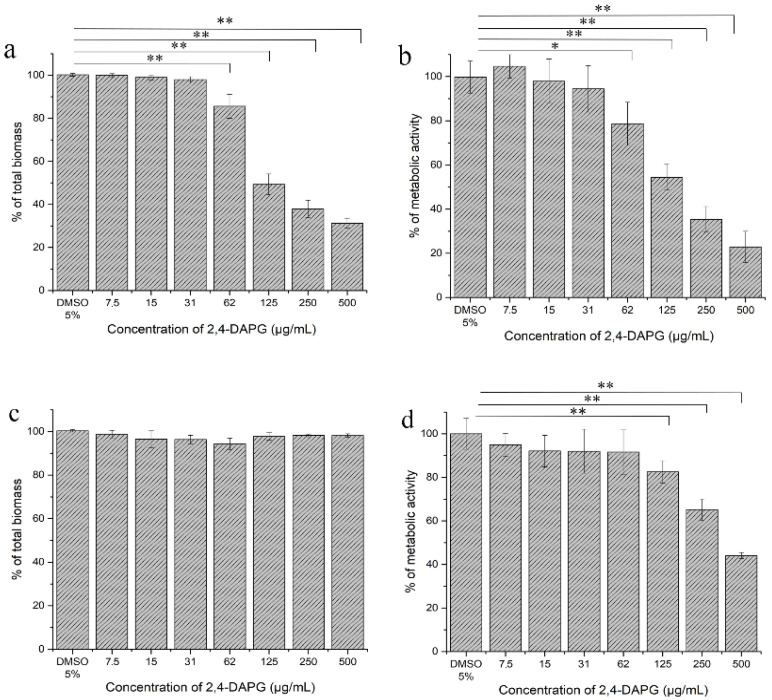
Influence of 2,4-DAPG on the *C. albicans* ATCC 10231 biofilm development (**a**,**b**) and on the mature biofilm (**c**,**d**). 2,4-DAPG action was estimated by measuring biofilm biomass (**a**,**c**) and metabolic activity of the biofilm-embedded cells (**b**,**d**). The y-axis represents percentages calculated with respect to the control sample. The means plus standard deviations of results from two independent experiments with three technical replicates are shown. Values that are significantly different from the values for “DMSO 5%” are indicated * *p* < 0.05, ** *p* < 0.01 (pair-sample Student’s *t*-test).

**Effects of 2,4-DAPG on the formation of *C. albicans* biofilm.** The effect of 2,4-DAPG on the biofilms was evaluated at concentrations corresponding to the minimum inhibitory concentration (MIC) for planktonic cells (MIC—125 µg/mL) (Figure S1a). It was found that the *C. albicans* biofilm was more resistant to 2,4-DAPG than the planktonic cells. 2,4-DAPG was unable to completely inhibit biofilm development at any used concentration, but it was able to reduce biofilm biomass and metabolic activity. 2,4-DAPG at 125 µg/mL halved the biofilm biomass at the end of the 48-h co-incubation (Figure 1a). The assessment of biofilms with redox indicator (TTC) indicated that the metabolic activity of the biofilm-embedded *Candida* cells decreased, which correlated with the reduction in the biofilm biomass (Figure 1b).

**Effects of 2,4-DAPG on the pre-formed *C. albicans* biofilm**. 2,4-DAPG was shown to affect the *C. albicans* pre-formed biofilms. 2,4-DAPG at the concentrations of 125 and 250 μg/mL reduced the metabolic activity of biofilm-embedded cells by 17.5 ± 5.2 and 34.8 ± 4.9%, respectively (Figure 1d). A more pronounced inhibition of the metabolic activity of the *Candida* cells (by 56%) was reached when the mature biofilm was exposed to 500 μg/mL of 2,4-DAPG. However, the eradication effect was not revealed at all taken concentrations (Figure 1c).

### 3.2. Microscopy Investigations of C. albicans Biofilms

**Figure 2:**
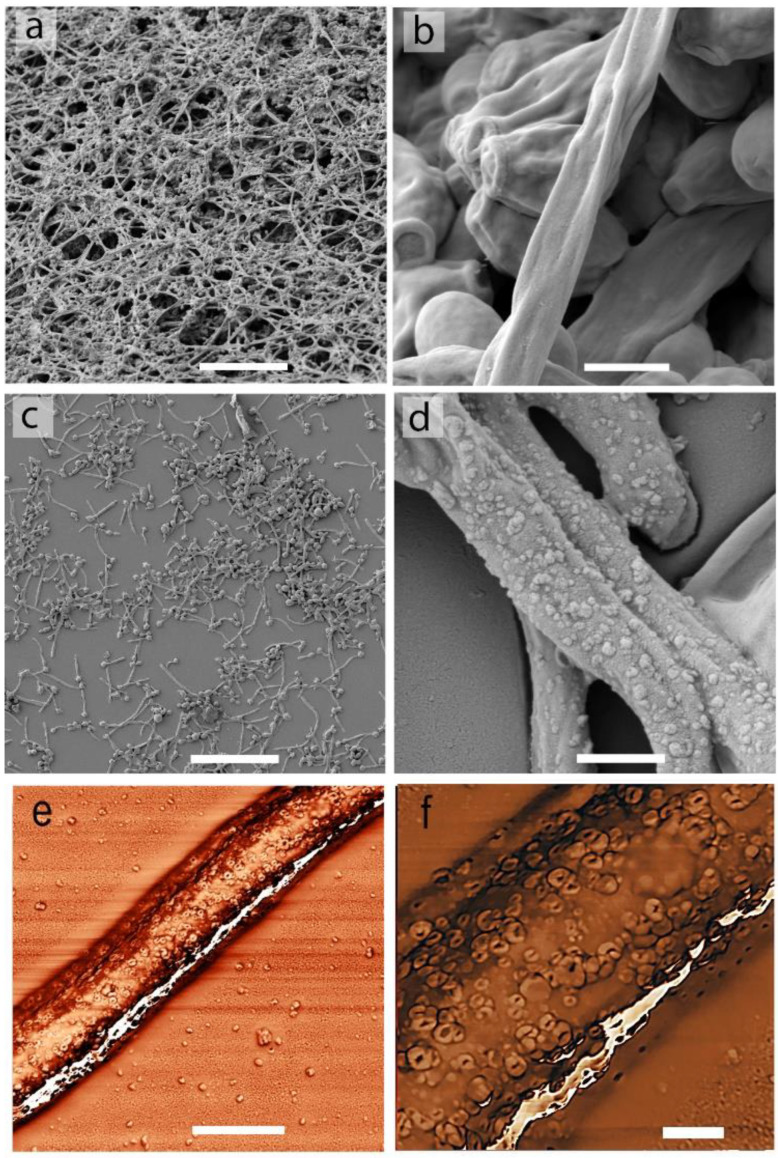
Microscopy investigations of *C. albicans* biofilm. Representative SEM images of the intact biofilm structure (**a**,**b**). SEM images of the biofilm formed in the presence of 2,4-DAPG (250 µg/mL) (**c**,**d**). Representative AFM images (phase shift mode) of hyphae treated with 2,4-DAPG (250 µg/mL) (**e**) and enlarged area of hyphae surface with protrusions (**f**). Scale bars—50 µm (**a**,**c**); 2 µm (**b**,**d**,**e**); 200 nm (**f**).

The biofilms were formed on the surface of glass slides in the presence of various concentrations of 2,4-DAPG and studied by scanning electron microscopy (SEM). Microscopic examination revealed some interesting features of biofilms treated with 2,4-DAPG. The main feature was related to the thickness of biofilms and their structure. Under the control condition (without 2,4-DAPG), the *Candida* cells formed long hyphae that intertwined with each other, forming a multilayer thickened biofilm (Figure 2a), while the treated biofilms (125–250 μg/mL) were visualized as a monolayer of adherent cells with short hyphae, which were spatially distributed on a glass surface (Figure 2c). The inhibition of biofilm formation was strongly dependent on 2,4-DAPG concentrations (Figure S2). 

A more detailed study of the cell morphology revealed some tiny differences between the normal (Figure 2b) and treated *Candida* biofilms. It was shown that hyphae, but not budding cells, contain numerous objects on their surface (Figure 2d). To exclude any SEM-related artifacts, we performed a study of the biofilms with atomic force microscopy (AFM). It turned out that the surfaces of *C. albicans* hyphae, which were grown in the presence of 2,4-DAPG (125–250 µg/mL), were decorated (Figure 2e) with numerous protrusions with invagination in their center (Figure 2f). The average diameter of the protrusions was 172 ± 42.6 nm. 

### 3.3. Chemical Analysis of C. albicans Biofilm’ Matrix

**Figure 3:**
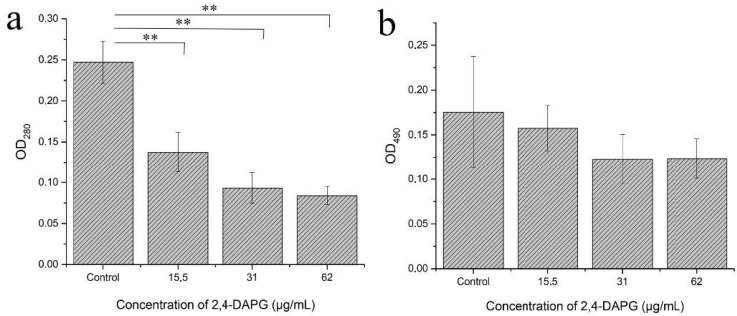
Protein (**a**) and carbohydrate content (**b**) in EPS of *C.albicans* biofilms which were formed under the 2,4-DAPG treatment for 24 h. The y-axis represents absorbance units. The means plus standard deviations of results from two independent experiments with three technical replicates are shown. Values that are significantly different from the values for “control” are indicated ** *p* < 0.01 (pair-sample Student’s *t*-test).

To fully understand the effect of the 2,4-DAPG treatment on the biofilm structure, the chemical analysis of extracellular polymeric substances (EPS) was performed. It was found that the treatment of the biofilm with sub-inhibitory concentrations of 2,4-DAPG led to dose-dependent reductions in the protein amount (Figure 3a), while the number of carbohydrates was not significantly altered compared to the control (Figure 3b).

### 3.4. Investigation of the Mechanisms Underlying the 2,4-DAPG Biofilm Inhibitory Activity

**Figure 4:**
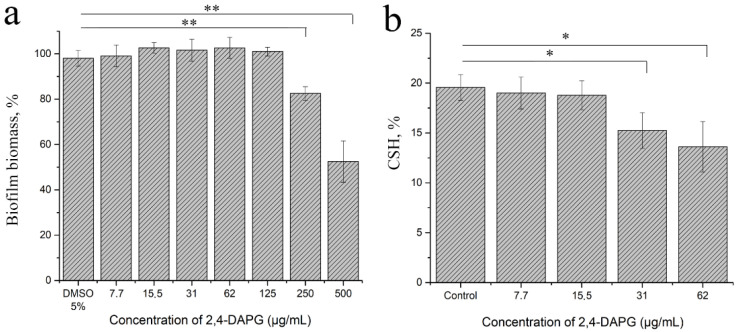
Total biomass quantification (crystal violet) assay was applied for estimation of the adhesion efficacy within 1.5 h (**a**). Adhesion to polystyrene and cellular surface hydrophobicity (CSH) of *C. albicans* ATCC 10231 cells (**b**). The means plus standard deviations of results from two independent experiments with three technical replicates are shown. Values that are significantly different from the values for “DMSO 5%” or “control” are indicated * *p* < 0.05, ** *p* < 0.01 (pair-sample Student’s *t*-test).

**Influence of 2,4-DAPG on adhesion and surface hydrophobicity of *C. albicans* cells.** The assessment of the ability of planktonic cells to adhere to the surface of a solid substrate revealed a reduction in the number of adherent cells by 17.4 ± 9.2 and 47.5 ± 3.0% when 2,4-DAPG was applied for 250 and 500µg/mL (Figure 4a). The concentrations of 2,4-DAPG less than 250 µg/mL did not affect the adhesion. At the same time, 2,4-DAPG at 30 and 60 μg/mL lowered the surface hydrophobicity of *Candida* cells by 20.0 ± 5.6 and 30.3 ± 2.6%, respectively (Figure 4b).

### 3.5. Effect of Sub-Inhibitory Concentration of 2,4-DAPG on the Aspartyl Protease and Lipase Activities of Planktonic and Biofilm of C. albicans

**Figure 5:**
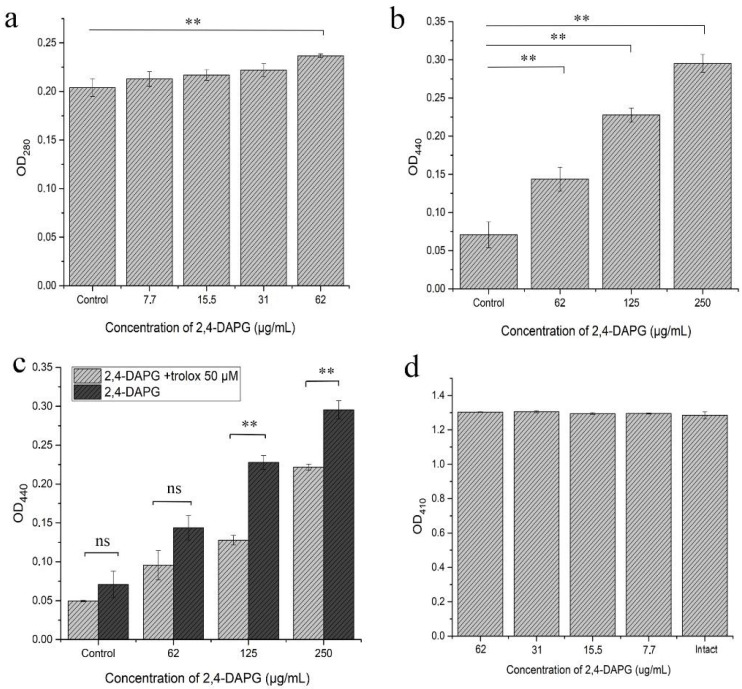
The protease activity of planktonic cells (Sap1–3) (**a**) and biofilms (Sap4–6) (**b**) of *C. albicans* ATCC 10231 under the influence of different concentrations of 2,4-DAPG. Trolox addition (50 µM) reduced Sap production (**c**). Lipase production was not affected by the action of 2,4-DAPG (**d**). The means plus standard deviations of results from two independent experiments with three technical replicates are shown. Values that are significantly different from the values for “control” are indicated ** *p* < 0.01, ns—values are not significantly different (pair-sample Student’s *t*-test).

The revealed protrusion-like objects on the hyphal surface led us to investigate which metabolites might be associated with this phenomenon. We found that the sub-inhibitory concentration of 2,4-DAPG (62 µg/mL) significantly increased the production of aspartyl proteases subfamily 1–3 (Sap1–3) (Figure 5a).

The enhancement of aspartyl protease activity in the planktonic cells of *C. albicans* treated with 2,4-DAPG encouraged us to check its secretion in biofilms. Sap1–3 activity was not detected in either 2,4-DAPG-treated biofilms or control samples. However, it was shown that the Sap4–6 activity of the *C. albicans* biofilms changed dose-dependently. Adding 2,4-DAPG at 62, 125 and 250 µg/mL increased the activity for 54.4 ± 3.0, 68.2 ± 2.7, and 75.8 ± 5.9%, respectively (Figure 5b). Adding antioxidant Trolox simultaneously with 2,4-DAPG significantly reduced the Sap4–6 activity of *C. albicans* biofilms (Figure 5c) without affecting the 2,4-DAPG antifungal property (Figure S1b). At the same time, the sub-inhibitory concentrations of 2,4-DAPG did not affect the lipase activity of *C. albicans* ATCC 10231 (Figure 5d).

### 3.6. Effect of 2,4-DAPG on C. albicans Dimorphism (Germ Tube Formation/Yeast-to-Hyphae Transition)

**Figure 6:**
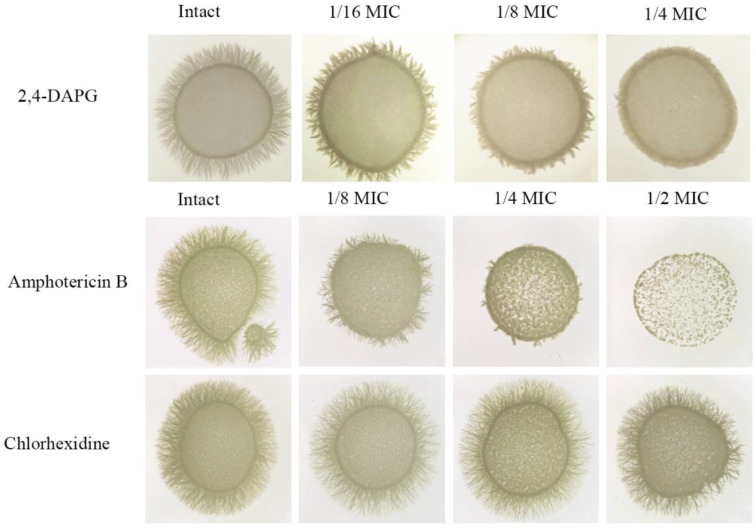
Yeast-to-hyphae transition of *C. albicans* ATCC 10231 on spider agar in the presence of sub-inhibitory concentrations of various antifungals. 2,4-DAPG was added at 31 µg/mL (1/4 MIC); 15.5 µg/mL (1/8 MIC); 7.5 µg/mL (1/16 MIC); and 0 µg/mL.

2,4-DAPG affected the morphogenesis of *Candida* cells by suppressing the yeast-to-hyphae transition process. Hyphae formation in the agar medium was completely inhibited when 2,4-DAPG was added at 31 µg/mL (1/4 MIC) and halved at a concentration of 15.5 µg/mL (1/8 MIC) (Figure 6). 

Amphotericin B was added at 0.15 µg/mL (1/2 MIC), 0.075 µg/mL (1/4 MIC) and 0,037 µg/mL (1/8 MIC); chlorhexidine was added at 0.0003% (1/2 MIC), 0.00015% (1/4 MIC) and 0.00007 (1/8 MIC). 

Similar results were obtained with amphotericin B (Figure 6). The observed suppression of *C. albicans* cell morphogenesis was unlikely due to the toxic stress since chlorhexidine bigluconate taken at sub-inhibitory concentrations did not affect the hyphal growth (Figure 6). 

The effect of 2,4-DAPG on the morphogenesis of *C. albicans* in a liquid medium was also evaluated. Adding 2,4-DAPG at sub-inhibitory concentrations completely inhibited (31 µg/mL) or significantly reduced (15.5 µg/mL) the germ tube formation (Figure S3).

### 3.7. 2,4-DAPG Affects Expression Level of Genes Responsible for the Production of Proteases and Filamentous Growth of C.albicans

**Figure 7:**
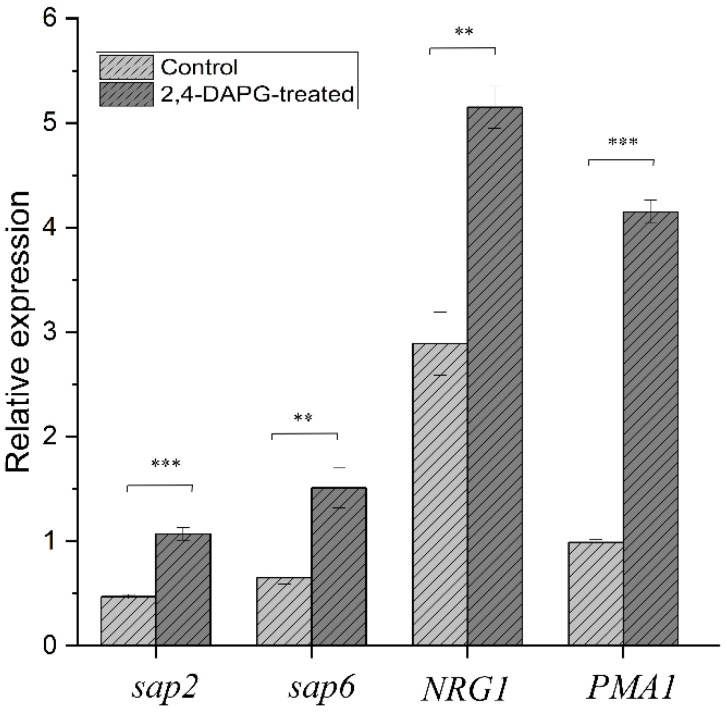
The analysis of the expression level of genes responsible for protease production and hyphae filamentation. Transcript levels of the analyzed genes were determined by RT-qPCR in relation to the *ACT1* expression. *Sap2*—the gene responsible for the synthesis of Sap 1–3 proteases; *Sap6*—the gene responsible for the synthesis of Sap4–6 proteases; *NRG1*—the negative regulator of filamentous growth; *PMA1*—the gene of H^+^ATPase. The means plus standard deviations of results from three technical replicates are shown. Values that are significantly different from the values for “control” are indicated **—*p* < 0.01; ***—*p* < 0.001 (pair-sample Student’s *t*-test).

The stress-response of *Candida* cells to the treatment was evaluated using real-time quantitative PCR analysis. It was found that the addition of 125 µg/mL of 2,4-DAPG to the growth medium significantly increased the relative expression of the *sap2* gene (2.27-fold) as well as the *sap6* gene (3.12-fold) (Figure 7). The expression level of the *NRG1* gene, a negative regulator of filamentation, was 1.75 and 1.89 times higher in the treated samples than in the control one (Figure 7). 

The relative expression of the *PMA1* gene, which encodes the *Candida* plasma membrane H^+^ATPase, was increased 4.2-fold in the 2,4-DAPG-treated sample (Figure 7).

### 3.8. Influence of 2,4-DAPG on the Production of Quorum Sensing Autoinducers

**Figure 8:**
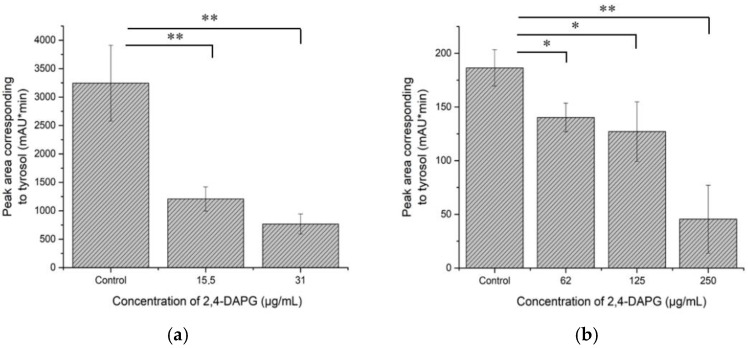
The amount of tyrosol which was produced by planktonic cells of *C. albicans* (**a**) and biofilms (**b**). The means plus standard deviations of results from two independent experiments with three technical replicates are shown. Values that are significantly different from the values for “Control” are indicated *—*p* < 0.05; **—*p* < 0.01 (pair-sample Student’s *t*-test).

Tyrosol is a molecule that is involved in quorum sensing controlled processes of *Candida* cell filamentation. It was found that the co-incubation of planktonic *Candida* cells with sub-inhibitory concentrations of 2,4-DAPG led to a dose-dependent decrease in tyrosol production (Figure 8a). 2,4-DAPG in 15.5 and 31.0 μg/mL reduced tyrosol production by 62.8 ± 6.5 and 76.8 ± 5.3%, respectively, compared to the control sample. 

The co-incubation of forming biofilms with inhibitory and sub-inhibitory concentrations of 2,4-DAPG (62–250 µg/mL) also led to a decrease in tyrosol production (Figure 8b). 2,4-DAPG at 62, 125, and 250 μg/mL has reduced tyrosol production by 25 ± 7.1, 32 ± 14.8, and 76 ± 16.7%, respectively. 

In this regard, we have evaluated the effect of the addition of the exogenous tyrosol on the ability of 2,4-DAPG to suppress hyphae formation. Using various combinations of tyrosol and 2,4-DAPG in the ‘checkerboard’ assay, we have failed to detect the restoration effect of exogenous tyrosol on the filamentation of *C. albicans* (Table S1).

## 4. Discussion 

2,4-DAPG is a secondary metabolite produced by a wide variety of *Pseudomonas* species [27]. In nature, the function of this molecule has not been reliably determined, but it is known that 2,4-DAPG production determines the properties of the rhizospheric pseudomonads as agents of biological control of phytopathogens [28,29,30].

It is also known that the antifungal effect of 2,4-DAPG on yeast cells is realized through the disruption of the mitochondrial membrane potential and the uncoupling of oxidative phosphorylation [4]. The determined range of the minimum fungicidal concentration on planktonic yeast cells is 60–120 µg/mL depending on the medium composition [3], which agrees with our results. At the same time, we found that adhered *Candida albicans* ATCC 10231 cells are significantly more resistant to 2,4-DAPG than planktonic cells. Perhaps the adhesion to surfaces leads to significant restructuring of *Candida* metabolism. Most likely, this restructuring is not specific to 2,4-DAPG since it is known that adhered *Candida* cells are more resistant to amphotericin B and azoles than its planktonic cells [31,32,33]. 

We have analyzed the changes in properties of treated *Candida* cells in order to deepen our understanding of the 2,4-DAPG biofilm inhibition mechanisms. It has been found that there is a significant reduction in cell surface hydrophobicity when *Candida* cells contact with 2,4-DAPG. Surface hydrophobicity is an important biophysical property of cells related to surface adhesion and biofilm formation [34,35]. 

Another finding concerns the prevention of the yeast–hyphal transition process. This process is directly related to the virulence of *C. albicans* and the formation of biofilms. The search for substances that is able to reduce virulence without a fungicidal effect is a perspective strategy for combating pathogens since it promises to achieve a therapeutic effect without resistance development [36,37]. Numerous works are dedicated to searching for microbial or plant-derived molecules that inhibit hyphal formation and transform *C. albicans* into a nonvirulent phenotype [38]. Our research demonstrated that the yeast–hyphal transition was disturbed when *Candida* cells were grown in a 2,4-DAPG medium. We have shown that 2,4-DAPG has the ability to inhibit hyphal formation at sub-inhibitory concentrations, while yeast colony growth has been unaffected. The inhibition of filamentation is accompanied by an increase in the expression of the *NRG1* gene—one of the negative transcriptional regulators of *Candida* filamentation [39]. 

Moreover, we found morphological changes in *Candida albicans* hyphae that occurred upon the treatment with 2,4-DAPG. 2,4-DAPG at concentrations of 125–250 μg/mL caused the formation of protrusions on the hyphal surface. Similar formations, called ”pimples”, were first described by Anderson et al. [40] in *C. albicans* WO-1 isolated from an immunosuppressed patient. Enzyme-containing channels having a similar morphology were then described in *C. albicans* when they were grown in a medium containing hydrocarbons [41]. The possible function of such channels (or protrusions) is the efflux of various biomolecules, including extracellular vesicles, which are involved in yeast virulence. Anderson et al. showed that *C. albicans* with pimples on the cell surface, as well as vesicles hiding under them, secrete at least 10 times more aspartyl protease than phenotype without pimples [40]. Aspartyl proteases constitute the main proteinase family directly involved in the pathogenesis of *Candida* species [42].

We have checked the aspartyl protease activity in the *C. albicans* biofilm after the 2,4-DAPG treatment. It was found that the increase in 2,4-DAPG concentration up to 125–250 µg/mL (by which channels were visualized) led to increased activity of hyphae-associated Sap4–6 proteases. Moreover, the increased expression level of the *sap4* gene supports this finding. Thus, the formation of channels on the surface of hyphae and the increase in the Sap4–6 activity at the same concentrations are interrelated phenomena. 

What is the trigger for the reduced yeast-to-hyphae transition and the increased production of aspartyl proteases under the influence of sub-inhibitory concentrations of 2,4-DAPG? 

A significant role in hyphal formation is assigned to quorum sensing. The regulation of *C. albicans* quorum sensing is mediated by small molecules tyrosol and farnesol, whose biosynthesis started at the log-phase and stationary phase, respectively [26,43,44]. In our study, we found a significant decrease in the tyrosol biosynthesis in *Candida* cells treated with 2,4-DAPG; however, it was not accompanied by a decrease in hyphal formation since exogenous tyrosol did not restore germ tube formation. A possible explanation can be found in the work of Grahl et al. [45]. The authors using metabolomic analysis of *C.albicans* metabolites, revealed a decrease (0.11–3.54-fold) in tyrosol production when the fungal cells were treated with phenazines, which disturb the respiration of cells [45]. Thus, quorum quenching is unlikely to be the mechanism that explains the inhibition of hyphae formation. 

The primary mode of action of 2,4-DAPG is the disruption of cellular (cytoplasmic) and mitochondrial membranes [3,4], leading to the disturbance of transmembrane potential and the malfunction of H^+^ATPase [46]. Disrupting the function of H^+^ATPase lead to the acidification of the cytoplasm [47]. We performed real-time PCR with total RNA isolated from the 2,4-DAPG-treated biofilm and revealed an increase in the expression of the *PMA1* gene, which indicates a violation of the work of H^+^ATPase [48]. Research shows that overexpression of *PMA1* as well as inhibition of H^+^ATPase by chemical inhibitor, results in a decrease in hyphal formation on inducing media (Spider, RPMI-1640) [49]. Along with H^+^ATPase, 2,4-DAPG affects the function of V-ATPase, since *S. cerevisiae* V-ATPase assembly and biosynthesis-related mutants were more sensitive to 2,4-DAPG [3]. The reduced synthesis and accumulation of ATP by mitochondria lead to a nutrient deficiency. These events affect *C. albicans* morphogenesis [45,50,51], but stimulated extracellular secretion of aspartyl proteases [52,53,54]. At the same time, 2,4-DAPG acts as a proton ionophore and dissipates the proton gradient across the mitochondrial membrane, triggering the production of reactive oxygen species [3,4].

Oxidative stress is known to have direct and indirect effects on eukaryotic cells. The direct action of reactive oxygen species oxidizes intracellular molecules (e.g., proteins, DNA, lipids of plasma membrane), while the indirect action regulates gene expression through various regulatory systems [55]. ROS sensors become activated, which leads to the adaptation of yeast to oxidative stress and to the activation of the genes responsible for the synthesis of aspartyl proteases and their secretion into extracellular environment [56]. 

It is well known that the production of aspartyl proteases could be increased by osmotic and oxidative stress as well as antifungal treatment, which have common transcriptional pathways [57,58,59,60].

If the production of reactive oxygen species acts as a trigger for Sap production, then the administration of antioxidants should affect the proteolytic activity of *Candida* cells. We used Trolox, a water-soluble analog of vitamin E that exhibits antioxidant activity. We found that adding Trolox simultaneously with 2,4-DAPG significantly reduces the Sap4–6 activity of *C. albicans* biofilms. 

## 5. Conclusions

Thus, in the present work, we have shown two aspects of the 2,4-DAPG action on *C. albicans.* We have demonstrated that 2,4-DAPG can affect the virulence of *C. albicans* due to reducing biofilm formation and hypha development. However, the production of proteolytic enzymes, which is the manifestation of the sub-inhibitory effect may have adverse effects on the host. ROS generation accompanies the antifungal action of 2,4-DAPG. One of the possible solutions to overcome the sub-inhibitory effect is the use of antioxidants in pair with 2,4-DAPG. The reduction or interruption of the intracellular signal triggered by ROS prevents arising undesirable fungal stress-response. All of these findings are important and could be useful in developing strategies for the treatment of diseases caused by *Candida albicans*.

## Data Availability

Not applicable.

## Supplementary Materials

The following supporting information can be downloaded at: https://www.mdpi.com/article/10.3390/jof8101018/s1, Figure S1: The growth curves of planktonic *C. albicans* ATCC 10231 cells; Figure S2: The scanning electron images of *C. albicans* ATCC 10231 biofilms; Figure S3. Inhibition of the *C. albicans* ATCC 10231 filamentation; Table S1: The ability of *Candida albicans* cells to form germ tube in the presence of 2,4-DAPG and exogenous tyrosol; Table S2. Primers used in the RT-qPCR study.
