# Supplementary material for: 2,4-Diacetylphloroglucinol Modulates Candida albicans Virulence
Source: J Fungi (Basel). 2022 Sep 27;8(10):1018. doi: 10.3390/jof8101018 (PMC9604888; doi:10.3390/jof8101018)
Supplement: Supplementary file 1 [file jof-08-01018-s001.zip › jof-1771039-supplementary.pdf]

2,4-Diacetylphloroglucinol Modulates *Candida albicans* Virulence

Artyom A. Stepanov. Darya V. Poshvina, and Alexey S. Vasilchenko*

Laboratory of antimicrobial resistance, Institute of Environmental and Agricultural Biology (X-BIO), Tyumen State University, Volodarskogo street, 6, 625003 Tyumen, Russia
* Correspondence: avasilchenko@gmail.com

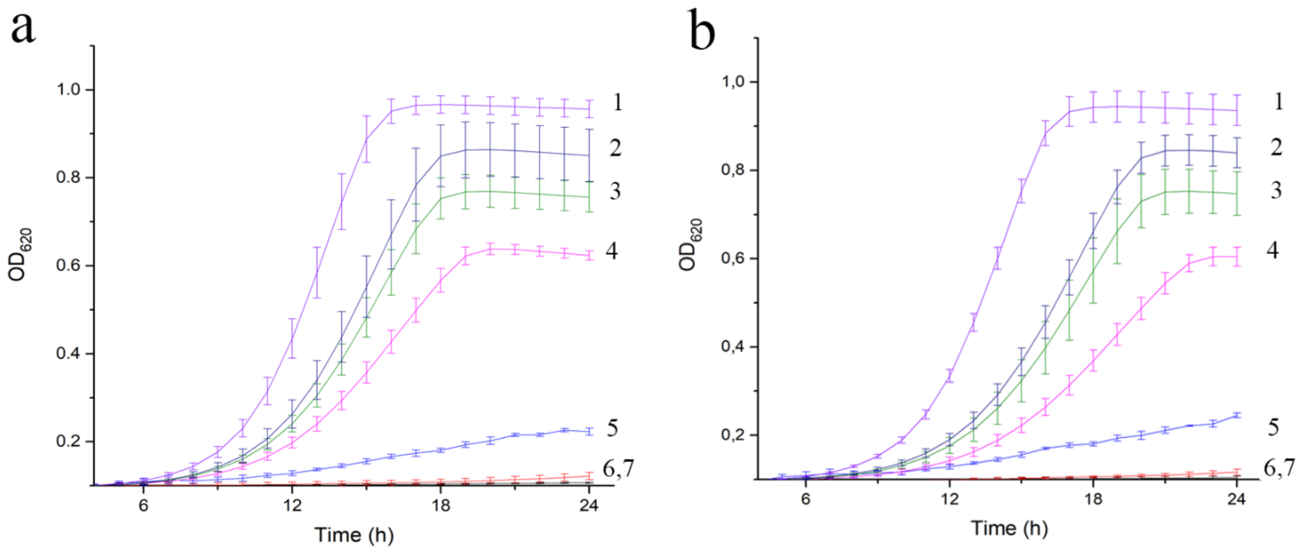

Figure S1. The growth curves of planktonic *C. albicans* ATCC 10231 cells. The assessment of 2,4-DAPG inhibitory activity was performed without addition of the antioxidant Trolox (**a**). and with adding for 50 μM (**b**). 2.4-DAPG was added for (μg/ml): 0 (1). 15.5 (2). 31 (3). 62 (4). 125 (5). 250 (6). 500 (7). Growth curves of *C. albicans* was built by recording the absorbance at 620 nm. The means plus standard deviations of the means of results from two independent experiments with three technical replicates are shown.

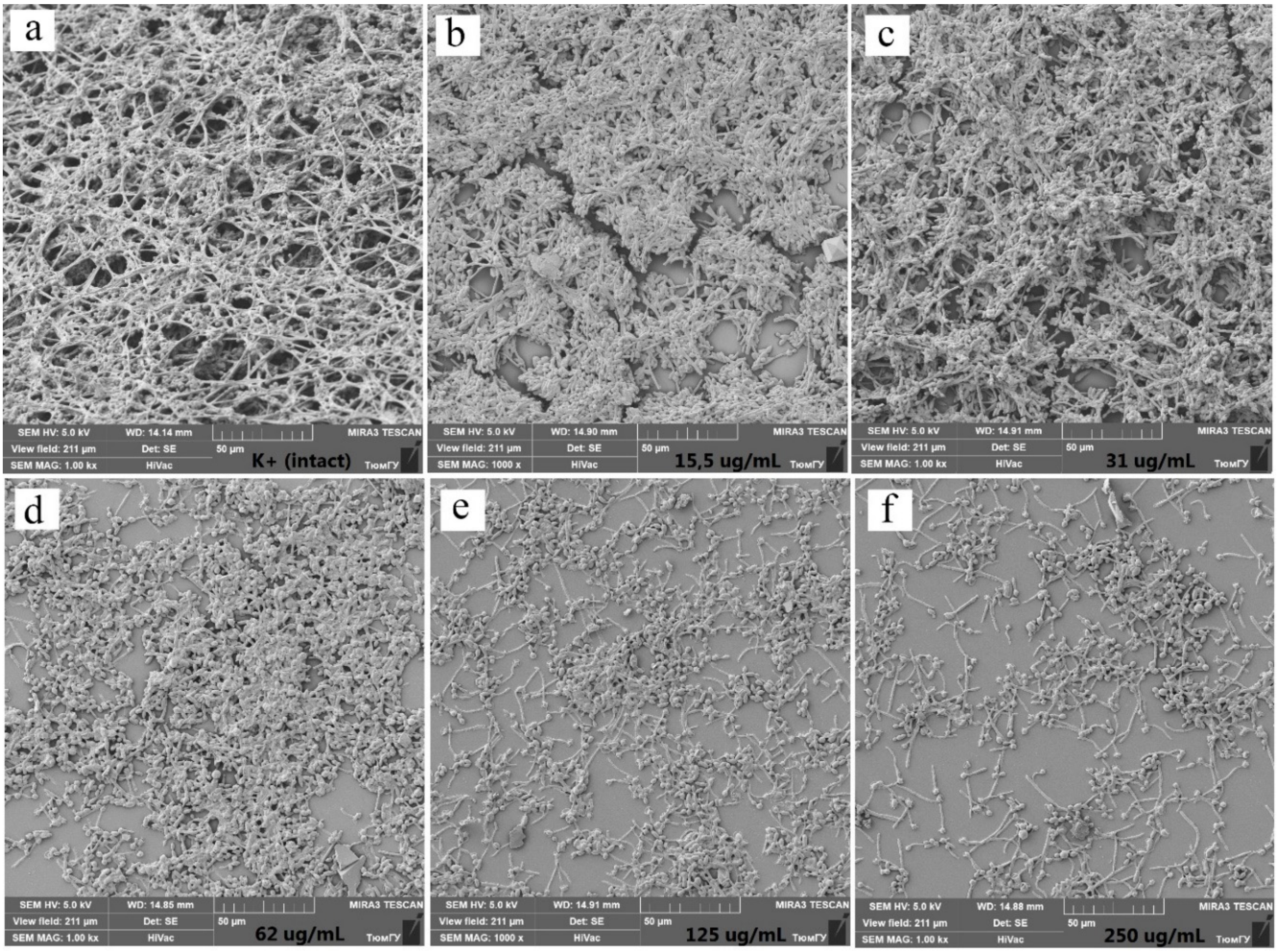

Figure S2. The scanning electron images of *C.albicans* ATCC 10231 biofilms. Biofilms were formed in the presence of 2.4-DAPG a - the control sample. b - 15.5 µg/mL. c- 31 µg/mL. d - 62 µg/mL. e - 125 µg/mL. f - 250 µg/mL.

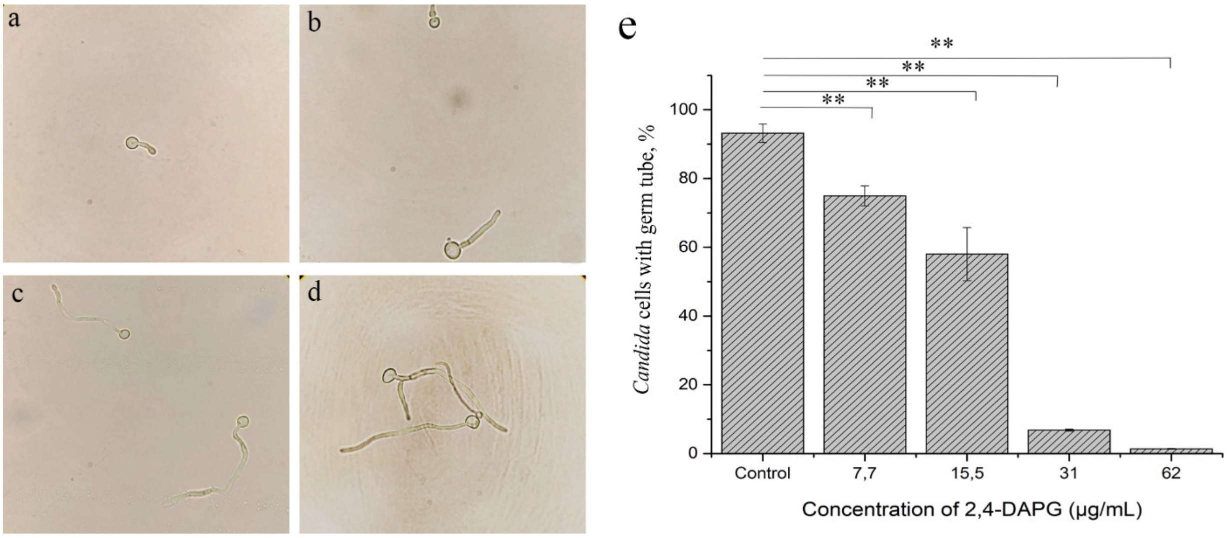

Figure S3. Inhibition of the *C. albicans* ATCC 10231 filamentation. The microscopic images show the morphology of *C. albicans* which grown for 3 h under the 2.4-DAPG treatment: a) 61 µg/mL (½ MIC); b) 30 µg/mL ¼ MIC; c) 15 µg/mL ⅛ MIC; d) Control (without the treatment). e) The percentage of *C. albicans* cells which formed a germ tube in the presence of different concentrations of 2.4-DAPG. The means plus standard deviations of the means of results from two independent experiments with three technical replicates are shown. Values that are significantly different from the values for "Control" are indicated * p <0.05, ** p<0.01 (pair-sample Student's t-Test).

Table S1. The ability of *Candida albicans* cells to form germ tube in the presence of 2,4-DAPG and exogenous tyrosol

		**2.4-DAPG, µg/mL**			
		31	15.5	7.7	0
Tyrosol, µM	100	3.75 ± 0.91	34.85 ± 3.88	77.7 ± 0.42	94.5 ± 0.14
	50	4.40 ± 1.83	42.35 ± 5.86	76.10 ± 3.67	93.25 ±0.21
	25	9.65 ± 0.77	44.30 ± 3.39	76.00 ± 2.26	93.55 ± 1.06
	12.5	7.25 ± 0.49	47.15 ± 3.18	77.02 ± 1.55	94.05 ± 1.76
	0	6.80 ± 0.42	50.55 ± 0.35	74.10 ± 4.10	96.93 ± 0.89

The checkerboard assay was performed using 2,4-DAPG and commercially available tyrosol. *Candida* cells were grown for 3 h in 96-wells plate. Then the samples were mounted on glass slides and microscopically evaluated. Percent of cells that formed germ tubes were calculated using formula of Okamoto et al. 1993. Data presents means ± SD (n – 300 = 100 fields of view per sample × 3 technical replicates).

Table S2. Primers used in the RT-qPCR study

**Gene description (designation)**	**Primer sequence**
Actin (*act1*)	F: CCAACTGGGACGATATGGAAA
	R: TTGGAGCTTCGGTCAACAA
Extracellular aspartic protease (*Sap2*)	F: CCAAGTGGTTCATCAGCTTCA
	R: ACCACCAAATCCAACGGTATC
Extracellular aspartic protease (*Sap6*)	F: GGAGAGTTAAGGAAGAGGGAATAAG
	R: GCCAACGAAGCTACCAGAA
Plasma membrane H+ATPase (*pma1*)	F: GATGCCGTTGGTATTGCTAAAG
	R: ACCACCGGACAAACCTAATC
Transcriptional repressor of hyphal development (*nrg1*)	F: CACCACCACCATATCCAATGA
	R: TGGCTGAGGTTGTTGTAGTG
